# Mitochondrial Genomes of Two Bombycoidea Insects and Implications for Their Phylogeny

**DOI:** 10.1038/s41598-017-06930-5

**Published:** 2017-07-26

**Authors:** Zhao-Zhe Xin, Yu Liu, Xiao-Yu Zhu, Ying Wang, Hua-Bin Zhang, Dai-Zhen Zhang, Chun-Lin Zhou, Bo-Ping Tang, Qiu-Ning Liu

**Affiliations:** Jiangsu Key Laboratory for Bioresources of Saline Soils, Jiangsu Synthetic Innovation Center for Coastal Bio-agriculture, Jiangsu Provincial Key Laboratory of Coastal Wetland Bioresources and Environmental Protection, School of Ocean and Biological Engineering, Yancheng Teachers University, Yancheng, 224051 PR China

## Abstract

The mitochondrial genome (mt genome) provides important information for understanding molecular evolution and phylogenetics. As such, the two complete mt genomes of *Ampelophaga rubiginosa* and *Rondotia menciana* were sequenced and annotated. The two circular genomes of *A*. *rubiginosa* and *R*. *menciana* are 15,282 and 15,636 bp long, respectively, including 13 protein-coding genes (PCGs), two rRNA genes, 22 tRNA genes and an A + T-rich region. The nucleotide composition of the *A*. *rubiginosa* mt genome is A + T rich (81.5%) but is lower than that of *R*. *menciana* (82.2%). The AT skew is slightly positive and the GC skew is negative in these two mt genomes. Except for *cox1*, which started with CGA, all other 12PCGs started with ATN codons. The A + T-rich regions of *A*. *rubiginosa* and *R*. *menciana* were 399 bp and 604 bp long and consist of several features common to Bombycoidea insects. The order and orientation of *A*. *rubiginosa* and *R*. *menciana* mitogenomes with the order *trnM*-*trnI*-*trnQ*-*nad2* is different from the ancestral insects in which *trnM* is located between *trnQ* and *nad2* (*trnI*-*trnQ*-*trnM*-*nad2*). Phylogenetic analyses indicate that *A*. *rubiginosa* belongs in the Sphingidae family, and *R*. *menciana* belongs in the Bombycidae family.

## Introduction

Insect mitochondrial DNA (mtDNA) is a double-stranded, circular molecule that is 14–19 kb in length and contains 13 PCGs: subunits 6 and 8 of the ATPase (*atp6* and *atp8*), cytochrome c oxidase subunits 1–3 (*cox1*–*cox3*), cytochrome B (*cob*), NADH dehydrogenase subunits 1–6 and 4 L (*nad1*–*6* and *nad4L*). It also contains two rRNA genes, small and large subunit rRNAs (*rrnL* and *rrnS*), 22 tRNA genes and a non-coding element termed the A + T-rich region^1^. The A + T-rich region has a higher level of sequence and length variability than other regions of the genome^2–5^ and regulates the transcription and replication of mt genomes^6^. As an informative molecular marker, mtDNA can provide important information for rearrangement patterns and phylogenetic analysis due to its rapid evolutionary rate and lack of genetic recombination^7^. Therefore, mtDNA has been widely used for diverse evolutionary studies among species^8^.

Recent advances in sequencing technologies have led to the rapid increase in mt genome data in GenBank, including Bombycoidea mt genomes. Bombycoidea is a superfamily of moths that contains the silk moths, emperor moths, sphinx moth, and relatives^9^. Some complete mt genomes of Bombycoidea insects are currently available in GenBank (Table 1). Several representative families were studied in this paper. Two families, Bombycidae and Saturniidae, are silk-producing insects with economic values in Bombycoidea^10^. The Sphingidae are a family of Bombycoidea, commonly known as hawk moths, sphinx moths, and hornworms; this family includes approximately 1,450 species^11, 12^. Brahmaeidae are a family of Bombycoidea^11, 12^. The Lasiocampidae are also a family of Bombycoidea, known as eggars, snout moths, or lappet moths. Over 2,000 species occur worldwide, and it is likely that not all have been named or studied^13^.

**Table 1:** List of Bombycoidea species analysed in this paper with their respective GenBank accession numbers.

Superfamily	Family	Species	Size (bp)	GBAN*
**Bombycoidea**	**Sphingidae**	***Ampelophaga rubiginosa***	**15**,**282**	**KT153024**
**Bombycoidea**	**Bombycidae**	***Rondotia menciana***	**15**,**636**	**KT258908**
Bombycoidea	Bombycidae	*Rondotia menciana*	15,301	KC881286
Bombycoidea	Bombycidae	*Rondotia menciana*	15,364	KJ647172
Bombycoidea	Bombycidae	*Andraca theae*	15,737	KX365419
Bombycoidea	Bombycidae	*Bombyx mandarina*	15,928	AB070263
Bombycoidea	Bombycidae	*Bombyx mori*	15,643	AF149768
Bombycoidea	Bombycidae	*Bombyx huttoni*	15,638	KP216766
Bombycoidea	Saturniidae	*Samia cynthia ricini*	15,384	JN215366
Bombycoidea	Saturniidae	*Actias selene*	15,236	JX186589
Bombycoidea	Saturniidae	*Antheraea pernyi*	15,566	AY242996
Bombycoidea	Saturniidae	*Antheraea yamamai*	15,338	EU726630
Bombycoidea	Saturniidae	*Eriogyna pyretorum*	15,327	FJ685653
Bombycoidea	Saturniidae	*Saturnia boisduvalii*	15,360	EF622227
Bombycoidea	Saturniidae	*Antheraea assama*	15,312	KU301792
Bombycoidea	Saturniidae	*Samia cynthia cynthia*	15,345	KC812618
Bombycoidea	Saturniidae	*Antheraea frithi*	15,338	KJ740437
Bombycoidea	Saturniidae	*Attacus atlas*	15,282	KF006326
Bombycoidea	Saturniidae	*Actias artemis aliena*	15,243	KF927042
Bombycoidea	Saturniidae	*Samia canningi*	15,384	KJ159909
Bombycoidea	Lasiocampidae	*Dendrolimus spectabilis*	15,411	KM244678
Bombycoidea	Lasiocampidae	*Dendrolimus tabulaeformis*	15,411	KJ913817
Bombycoidea	Lasiocampidae	*Dendrolimus punctatus*	15,411	KJ913813
Bombycoidea	Lasiocampidae	*Apatelopteryx phenax*	15,552	KJ508055
Bombycoidea	Lasiocampidae	*Trabala vishnou guttata*	15,281	KU884483
Bombycoidea	Lasiocampidae	*Euthrix laeta*	15,368	KU870700
Bombycoidea	Sphingidae	*Daphnis nerii*	15,247	
Bombycoidea	Sphingidae	*Agrius convolvuli*	15,349	
Bombycoidea	Sphingidae	*Manduca sexta*	15,516	EU286785
Bombycoidea	Sphingidae	*Sphinx morio*	15,299	KC470083
Bombycoidea	Sphingidae	*Notonagemia analis scribae*	15,303	KU934302
Bombycoidea	Brahmaeidae	*Brahmaea hearseyi*	15,442	KU884326

*GenBank accession number.

Here, we sequenced the complete mt genomes of two species, *A*. *rubiginosa* and *R*. *menciana*. We aimed to analyse the mt genomes of these two species and to investigate the phylogeny of Bombycoidea insects. We were particularly interested in the phylogenetic position of Sphingidae and Bombycidae based on the 32 Bombycoidea complete mt genomes available to date.

## Materials and Methods

### Specimen collection

The moths of *A*. *rubiginosa* and *R*. *menciana* were collected in Xuancheng, Anhui Province. Total DNA was isolated using the Genomic DNA Extraction Kit (SangonBiotech, China) according to manufacturer instructions. Extracted DNA was used to amplify the complete mt genomes by PCR.

### PCR amplification and sequencing

For amplification of the entire mt genomes of *A*. *rubiginosa* and *R*. *menciana*, specific primers were designed based on mt genomes sequences obtained from other Lepidopteran insects^14, 15^ (Table 2). The complete mt genomes were obtained using a combination of conventional PCR and long PCR to amplify overlapping fragments spanning the complete mt genomes. All amplifications were performed on an Eppendorf Mastercycler and Mastercycler gradient in 50 µl reaction volumes with 5 µl of 10 × Taq Buffer (Mg^2+^) (Aidlab), 4 µl of dNTPs (2.5 mM, Aidlab), 2 µl of each primer (10 µM), 2 µl of DNA (~100 ng), 34.5 µl of ddH_2_O, and 0.5 µl of Red Taq DNA polymerase (5U, Aidlab). PCR was performed under the following conditions: 3 min at 94 °C, followed by 35 cycles of 30 s at 94 °C, 1–3 min at 54–60 °C (depending on primer combination), elongation at 72 °C for 30 s to 4 min (depending on the fragment length) and final extension at 72 °C for 10 min. The PCR products were separated by agarose gel electrophoresis (1% w/v) and purified using a DNA gel extraction kit (Transgene, China). The purified PCR products were ligated into the T-vector (SangonBiotech, China) and sequenced.

**Table 2:** Primers used in this study.

Primer	Sequence (5′–3′)	Annealing temperature	Region
F1	GCTTTTGGGCTCATACCTCA	56 °C	*trnM*-*cox1*
R1	GATGAAATACCTGCAAGATGAAG		
F2	TGGAGCAGGAACAGGATGAAC	55 °C	*cox1*-*trnK*
R2	GAGACCADTACTTGCTTTCAG		
F3	ATTTGTGGAGCTAATCATAG	56 °C	*cox2*- *cox3*
R3	GGTCAGGGACTATAATCTAC		
F4	TCGACCTGGAACTTTAGC	55 °C	*atp6*- *nad5*
R4	GCAGCTATAGCCGCTCCTACT		
F5	TAAAGCAGAAACAGGAGTAG	54 °C	*nad5*
R5	ATTGCGATATTATTTCTTTTG		
F6	CCCCAGCAGTAACTAAAGTAGAAG	54 °C	*nad5*-*cob*
R6	GTTAAAGTGGCATTATCT		
F7	GGAGCTTCTACATGAGCTTTTGG	56 °C	*nad4*-*rrnL*
R7	GTTTGCGACCTCGATGTTG		
F8	GGTCCCTTACGAATTTGAATATATCCT	60 °C	*nad1*-*rrnS*
R8	AAACTAGGATTAGATACCCTATTAT		
F9	CTCTACTTTGTTACGACTTATT	55 °C	*rrnS*-*nad2*
R9	TCTAGGCCAATTCAACAACC		

### Sequence analysis

Annotation of sequences were performed using the blast tools in NCBI web site (https://blast.ncbi.nlm.nih.gov/Blast.cgi). The sequences were edited and assembled using EditSeq and SeqMan (DNAStar package, DNAStar Inc. Madison, WI, USA). The graphical maps of *A*. *rubiginosa* and *R*. *menciana* complete mt genomes were drawn using the online mitochondrial visualization tool mtviz (http://pacosy.informatik.uni-leipzig.de/mtviz). The nucleotide sequences of PCGs were translated with the invertebrate mt genome genetic code. Alignments of *A*. *rubiginosa* and *R*. *menciana* PCGs with various Bombycoidea mt genomes were performed using MAFFT^16^. Composition skewness was calculated according to the following formulas:

$${\rm{AT}}\,{\rm{skew}}=[{\rm{A}}-{\rm{T}}]/[{\rm{A}}+{\rm{T}}];\,{\rm{GC}}\,{\rm{skew}}\,=\,[{\rm{G}}-{\rm{C}}]/[{\rm{G}}+{\rm{C}}].$$

Nucleotide composition statistics and codon usage were computed using MEGA 5.0^17^.

### Phylogenetic analysis

Thirty complete Bombycoidea mt genomes were downloaded from GenBank (https://www.ncbi.nlm.nih.gov/genbank/). In addition, mt genomes of *Biston panterinaria* and *Phthonandria atrilineata* were downloaded from GenBank and used as outgroup taxa. GenBank sequence information is shown in Table 1.

We estimated the taxonomic status of *A*. *rubiginosa* and *R*. *menciana* within Bombycoidea by constructing phylogenetic trees. Sequences from the PCGs of 34 mt genomes were combined. Two inference methods were used for analysis: Bayesian inference (BI) and Maximum likelihood (ML). BI was performed with MrBayes v 3.2.1^18^. While ML was performed with raxmlGUI^19^. Nucleotide substitution model selection was done using the Akaike information criterion implemented in MrModeltest v 2.3^20^. ProtTest version 1.4^21^ was used to select the amino acid substitution model. The GTR + I + G model was the best for nucleotide data, and the MtREV + I + G + F model was the best for amino acids. ML analysis was performed on 1000 bootstrapped datasets. The Bayesian analysis ran as 4 simultaneous MCMC chains for 10,000,000 generations, sampled every 100 generations, with a burn-in of 5000 generations. Convergence was tested for the Bayesian analysis by ensuring that the average standard deviation of split frequencies was less than 0.01. Additionally, we tested for sufficient parameter sampling by ensuring an ESS of more than 200 using the software Tracer v1.6^22^. The resulting phylogenetic trees were visualized in FigTree v1.4.2^23^.

## Results and Discussion

### Genome structure, organization and composition

**Figure 1:**
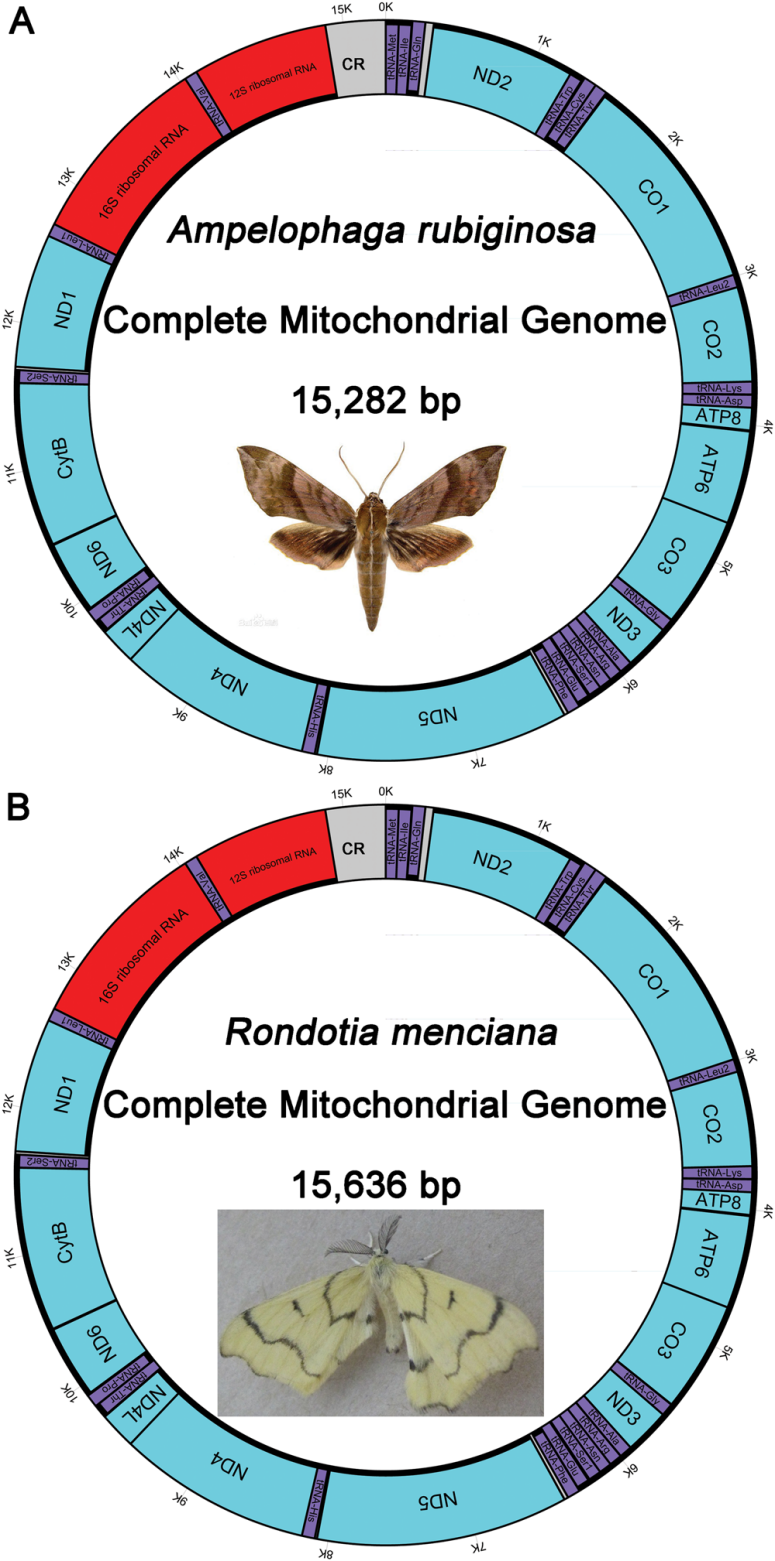
Circular map of the mt genomes of *A*. *rubiginosa* (**A**) and *R*. *Menciana* (**B**). *tRNA*-*Ser1*, *tRNA*-*Ser2*, *tRNA*-*Leu1* and *tRNA*-*Leu2* denote codons *tRNA*-*Ser1* (AGN), *tRNA*-*Ser2* (UCN), *tRNA*-*Leu1* (CUN), and *tRNA*-*Leu2* (UUR), respectively.

**Figure 2:**
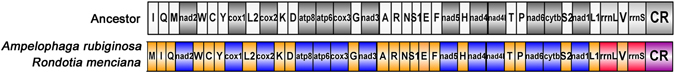
The mitochondrial gene order of ancestral insects and *A*. *rubiginosa and R*. *menciana*.

The complete sequences of *A*. *rubiginosa* and *R*. *menciana*, 15,282 bp and 15,636 bp in size, respectively, were determined and submitted to GenBank (Accession No. KT153024 and KT258908). These two mt genomes both contain 13 PCGs, two rRNA genes, 22 tRNA genes, and an A + T-rich region. Four of the 13 PCGs (*ND5*, *ND4*, *ND4L*, and *ND1*), 8 tRNAs (*trnQ*, *trnC*, *trnY*, *trnF*, *trnH*, *trnP*, *trnL* (CUN), and *trnV*) and two rRNAs (*rrnL* and *rrnS*) are coded with the minority-strand, while the remaining 23 genes are encoded by the majority-strand in *A*. *rubiginosa* and *R*. *menciana* (Fig. 1, Table 3). The length of the *R*. *menciana* mt genome (15,636 bp) is larger than *A*. *rubiginosa* (15,282 bp) and smaller than that of *Bombyx mandarina* (15,928 bp), *B*. *mori* (15,643 bp) and *B*. *huttoni* (15,638 bp), but it falls within the range (15,236–15,928 bp) of other known Bombycoidea mt genomes in our study (Table 1). The nucleotide composition of the *A*. *rubiginosa* mt genome is as follows (Table 4): A = 6,334 (41.4%), T = 6,126 (40.1%), G = 1,144 (7.5%), and C = 1,678 (11.0%). The nucleotide composition of the *A*. *rubiginosa* mt genome is A + T rich (81.5%) but is lower than that of *R*. *menciana* (82.2%). The AT skew^24^ is slightly positive and the GC skew is negative in these two mt genomes (Table 4), indicating an obvious bias towards the use of As and Cs. The order and orientation of genes in the *A*. *rubiginosa* and *R*. *menciana* mt genomes are identical to other bombicoid insects sequenced to date^25^, but differ from ancestral insects^26^. The placement of the *trnM* gene in the *A*. *rubiginosa* and *R*. *menciana* mt genome is *trnM*-*trnI*-*trnQ*, while in ancestral insects, it is *trnI*-*trnQ*-*trnM* (Fig. 2). Ghost moths exhibited the ancestral insect placement of the *trnM* gene cluster^27^. The hypothesis that the ancestral arrangement of the *trnM* gene cluster underwent rearrangement after Hepialoidea diverged from other Lepidopteran lineages was supported by our results in *A*. *rubiginosa* and *R*. *menciana*. The tRNA rearrangements are generally presumed to be a consequence of tandem duplication of partial mt genomes^28–31^, followed by random or non-random loss of the duplicated copies^28, 32, 33^.

**Table 3:** Summary of the mt genomes of *A*. *rubiginosa* and *R*. *menciana*.

Gene	Direction	Location	Size	Anticodon	Start codon	Stop codon	Intergenic nucleotides
*trnM*	F	1–68	68	CAT	—	—	0
*trnI*	F	69–132	64	GAT	—	—	−3
*trnQ*	R	130–198	69	TTG	—	—	56
*nad2*	F	255–1266	1012	—	ATT	T	0
*trnW*	F	1267–1334	68	TCA	—	—	−8
*trnC*	R	1327–1391	65	GCA	—	—	0
*trnY*	R	1392–1456	65	GTA	—	—	6
*cox1*	F	1463–2990	1528	—	CGA	T	0
*trnL2*(UUR)	F	2991–3058	68	TAA	—	—	0
*cox2*	F	3059–3740	682	—	ATG	T	0
*trnK*	F	3741–3811	71	CTT	—	—	2
*trnD*	F	3814–3881	68	GTC	—	—	0
*atp8*	F	3882–4043	162	—	ATT	TAA	−7
*atp6*	F	4037–4714	678	—	ATG	TAA	0
*cox3*	F	4715–5506	792	—	ATG	TAA	2
*trnG*	F	5509–5574	66	TCC	—	—	0
*nad3*	F	5575–5926	352	—	ATT	T	0
*trnA*	F	5927–5993	67	TGC	—	—	1
*trnR*	F	5995–6058	64	TCG	—	—	0
*trnN*	F	6059–6124	66	GTT	—	—	0
*trnS1*(AGN)	F	6125–6186	62	GCT	—	—	9
*trnE*	F	6196–6263	68	TTC	—	—	−2
*trnF*	R	6262–6327	66	GAA	—	—	27
*nad5*	R	6355–8076	1722	—	ATT	A	15
*trnH*	R	8092–8155	64	GTG	—	—	0
*nad4*	R	8156–9490	1335	—	ATG	TAA	0
*nad4L*	R	9491–9781	291	—	ATG	TAA	4
*trnT*	F	9786–9851	66	TGT	—	—	−1
*trnP*	R	9851–9916	66	TGG	—	—	6
*nad6*	F	9923–10,453	531	—	ATG	TAA	6
*cob*	F	10,460–11,608	1149	—	ATG	TAA	−1
*trnS2*(UCN)	F	11,608–11,672	65	TGA	—	—	21
*nad1*	R	11,694–12,629	936	—	ATG	TAA	0
*trnL1*(CUN)	R	12,630–12,696	67	TAG	—	—	0
*rrnL*	R	12,697–14,040	1344	—	—	—	0
*trnV*	R	14,041–14,108	68	TAC	—	—	0
*rrnS*	R	14,109–14,883	775	—	—	—	0
A + T-rich region		14,884–15,282	399	—	—	—	
*trnM*	F	1–68	68	CAT	—	—	0
*trnI*	F	69–132	64	GAT	—	—	−3
*trnQ*	R	130–198	69	TTG	—	—	52
*nad2*	F	251–1264	1014	—	ATT	TAA	7
*trnW*	F	1272–1338	67	TCA	—	—	−8
*trnC*	R	1331–1394	64	GCA	—	—	0
*trnY*	R	1395–1459	65	GTA	—	—	9
*cox1*	F	1469–2999	1531	—	CGA	T	0
*trnL2*(UUR)	F	3000–3066	67	TAA	—	—	0
*cox2*	F	3067–3748	682	—	ATG	T	0
*trnK*	F	3749–3819	71	CTT	—	—	−1
*trnD*	F	3819–3884	66	GTC	—	—	0
*atp8*	F	3885–4046	162	—	ATC	TAA	−7
*atp6*	F	4040–4717	678	—	ATG	TAA	3
*cox3*	F	4721–5509	789	—	ATG	TAA	2
*trnG*	F	5512–5577	66	TCC	—	—	0
*nad3*	F	5575–5931	357	—	ATA	TAA	27
*trnA*	F	5959–6032	74	TGC	—	—	10
*trnR*	F	6043–6105	63	TCG	—	—	0
*trnN*	F	6106–6173	68	GTT	—	—	6
*trnS1*(AGN)	F	6180–6248	69	GCT	—	—	1
*trnE*	F	6250–6314	65	TTC	—	—	3
*trnF*	R	6318–6385	68	GAA	—	—	0
*nad5*	R	6386–8124	1739	—	ATT	TA	0
*trnH*	R	8125–8190	66	GTG	—	—	10
*nad4*	R	8201–9541	1341	—	ATG	TAA	5
*nad4L*	R	9547–9837	291	—	ATG	TAA	2
*trnT*	F	9840–9904	65	TGT	—	—	0
*trnP*	R	9905–9970	66	TGG	—	—	2
*nad6*	F	9973–10,503	531	—	ATG	TAA	7
*cob*	F	10,511–11,665	1155	—	ATG	TAA	10
*trnS2*(UCN)	F	11,676–11,727	52	TGA	—	—	33
*nad1*	R	11,761–12,699	939	—	ATG	TAA	1
*trnL1*(CUN)	R	12,701–12,770	70	TAG	—	—	0
*rrnL*	R	12,771–14,186	1416	—	—	—	0
*trnV*	R	14,187–14,252	66	TAC	—	—	0
*rrnS*	R	14,253–15,032	780	—	—	—	0
A + T-rich region		15,033–15,636	604	—	—	—

**Table 4:** Composition and skewness in the *A*. *rubiginosa* and *R*. *menciana* mt genomes.

*A*. *rubiginosa*	Size (bp)	A (bp)	tCT (bp)	G (bp)	C (bp)	A %	T %	G %	C %	AT %	AT skew	GC skew
Whole genome	15,282	6334	616126	1144	1678	41.4	40.1	7.5	11.0	81.5	0.017	−0.189
Protein-coding genes	11,175	3894	5090	1135	1056	34.8	45.5	10.2	9.5	80.3	−0.133	0.038
tRNA genes	1461	602	589	116	154	41.2	40.3	7.9	10.6	81.5	0.011	−0.141
rRNA genes	2119	906	887	104	222	42.8	41.9	4.9	10.4	84.7	0.011	−0.362
A + T-rich region	399	174	194	14	17	43.6	48.6	3.5	4.3	92.2	−0.054	−0.097
Whole genome	15,636	6561	6290	1122	1663	42.0	40.2	7.2	10.6	82.2	0.021	−0.194
Protein-coding genes	11,205	3934	5107	1114	1050	35.1	45.6	9.9	9.4	80.7	−0.130	0.030
tRNA genes	1460	606	588	115	151	41.5	40.3	7.9	10.3	81.8	0.015	−0.135
rRNA genes	2196	959	927	100	210	43.7	42.2	4.5	9.6	85.9	0.017	−0.355
A + T-rich region	604	281	287	18	18	46.5	47.5	3.0	3.0	94.0	−0.011	0

### Protein-coding genes

**Figure 3:**
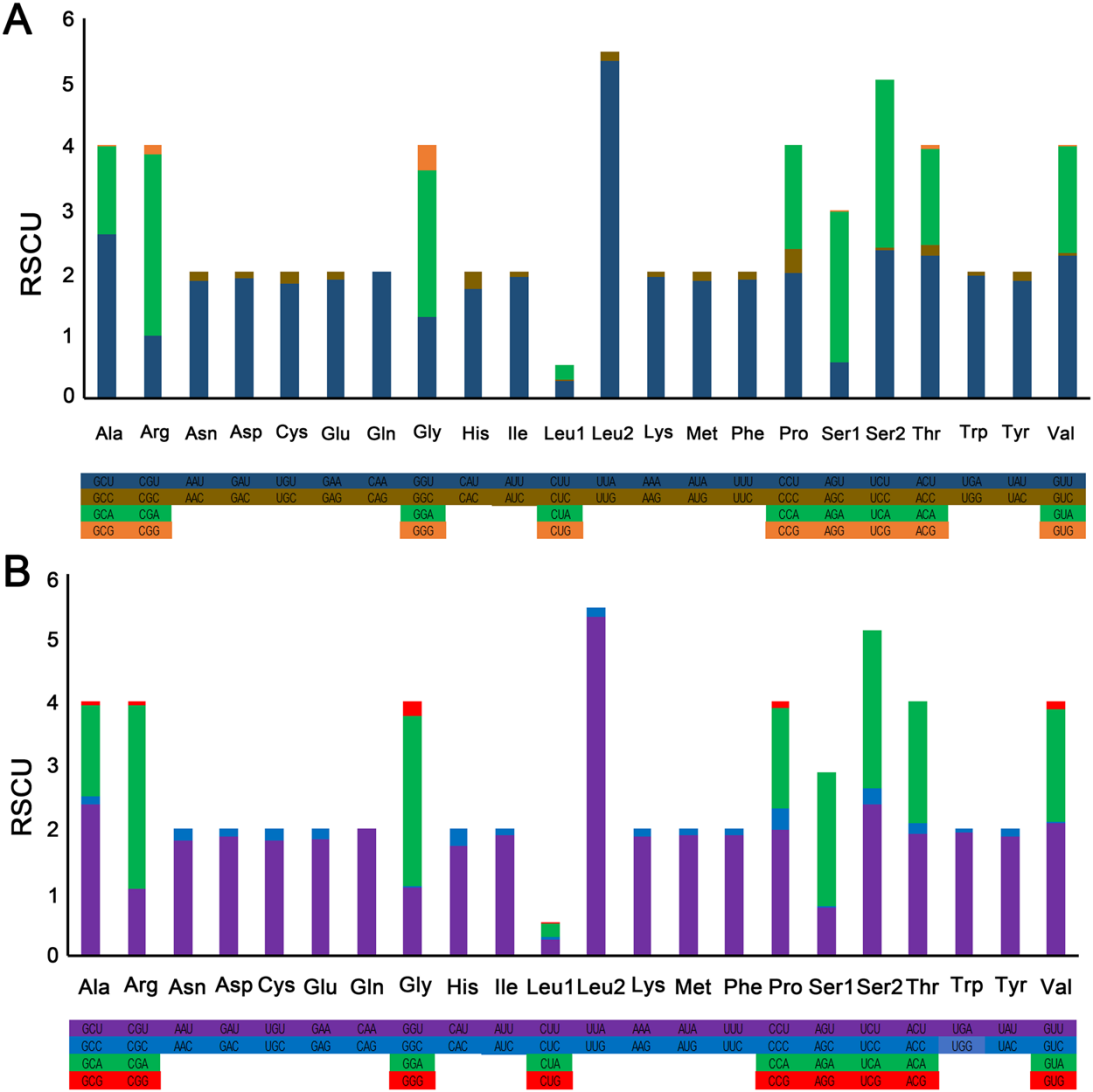
The relative synonymous codon usage (RSCU) in the mt genomes of *A*. *rubiginosa* (**A**) and *R*. *menciana* (**B**).

**Figure 4:**
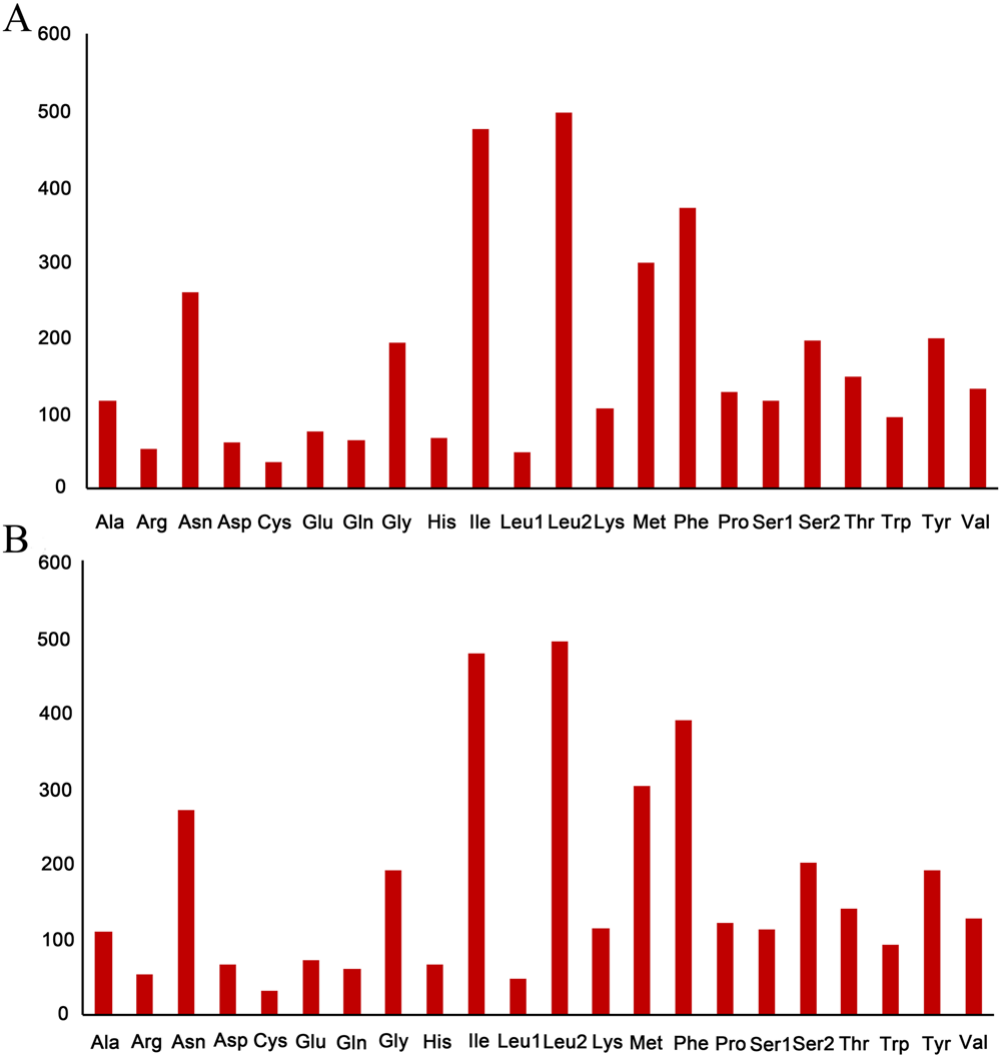
Amino acid composition in the mt genomes of *A*. *rubiginosa* (**A**) and *R*. *menciana* (**B**).

Summaries of the genes that make up the mt genomes of *A*. *rubiginosa* and *R*. *menciana* are given in Table 3. Twelve of the thirteen PCGs use standard ATN start codons in *A*. *rubiginosa* and *R*. *menciana*, except for *cox1*, which is initiated by the CGA codon (arginine). The CGA codon is highly conserved across most insect groups^14, 34^. In *A*. *rubiginosa*, eight PCGs (*atp8*, *atp6*, *cox3*, *nad4*, *nad4L*, *nad6*, *cob*, and *nad1*) have the complete stop codon TAA, while the remaining five terminate with either T (*nad2*, *cox1*, *cox2*, and *nad3*) or A (*nad5*). In *R*. *menciana*, ten PCGs (*nad2*, *atp8*, *atp6*, *cox3*, *nad3*, *nad4*, *nad4L*, *nad6*, *cob*, and *nad1*) have the complete stop codon TAA, while the remaining three terminate with either T (*cox1* and *cox2*) or TA (*nad5*). For *A*. *rubiginosa*, the average AT content of the 13 PCGs is 80.3%, and the overall AT and GC skews are –0.133 and 0.038, showing that T and G are more abundant than A and C. Similarly, the A + T composition of the 13 PCGs in the mt genome of *R*. *menciana* is 80.7%, while the AT and GC skews are –0.130 and 0.030, showing that T and G are more abundant than A and C (Table 4). Relative synonymous codon usage (RSCU) values for the *A*. *rubiginosa* and *R*. *menciana* mt genomes are summarized in Table 5 and Fig. 3, which show that NNT and NNA are more frequent than NNG and NNC, indicating a strong A or T bias in the third codon position. The most common amino acids for *A*. *rubiginosa* and *R*. *menciana* mitochondrial proteins are *Leu* (UUR), *Ile*, and *Phe* (Fig. 4).

**Table 5:** Codon number and RSCU in the *A*. *rubiginosa* and *R*. *menciana* mitochondrial PCGs.

Codon	Count	RSCU	Codon	Count	RSCU	Codon	Count	RSCU	Codon	Count	RSCU
UUU(F)	347	1.88	UCU(S)	91	2.35	UAU(Y)	184	1.86	UGU(C)	31	1.82
UUC(F)	23	0.12	UCC(S)	1	0.03	UAC(Y)	14	0.14	UGC(C)	3	0.18
UUA(L)	482	5.32	UCA(S)	103	2.66	UAA(*)	10	2	UGA(W)	91	1.94
UUG(L)	14	0.15	UCG(S)	0	0	UAG(*)	0	0	UGG(W)	3	0.06
CUU(L)	26	0.29	CCU(P)	63	1.98	CAU(H)	57	1.73	CGU(R)	13	1
CUC(L)	2	0.02	CCC(P)	12	0.38	CAC(H)	9	0.27	CGC(R)	0	0
CUA(L)	20	0.22	CCA(P)	52	1.64	CAA(Q)	63	2	CGA(R)	37	2.85
CUG(L)	0	0	CCG(P)	0	0	CAG(Q)	0	0	CGG(R)	2	0.15
AUU(I)	452	1.91	ACU(T)	83	2.26	AAU(N)	239	1.85	AGU(S)	22	0.57
AUC(I)	22	0.09	ACC(T)	6	0.16	AAC(N)	19	0.15	AGC(S)	0	0
AUA(M)	276	1.86	ACA(T)	56	1.52	AAA(K)	102	1.92	AGA(S)	92	2.37
AUG(M)	21	0.14	ACG(T)	2	0.05	AAG(K)	4	0.08	AGG(S)	1	0.03
GUU(V)	74	2.26	GCU(A)	75	2.59	GAU(D)	58	1.9	GGU(G)	62	1.29
GUC(V)	1	0.03	GCC(A)	0	0	GAC(D)	3	0.1	GGC(G)	0	0
GUA(V)	55	1.68	GCA(A)	40	1.38	GAA(E)	70	1.87	GGA(G)	111	2.31
GUG(V)	1	0.03	GCG(A)	1	0.03	GAG(E)	5	0.13	GGG(G)	19	0.4
UUU(F)	369	1.9	UCU(S)	94	2.39	UAU(Y)	178	1.87	UGU(C)	29	1.81
UUC(F)	20	0.1	UCC(S)	10	0.25	UAC(Y)	12	0.13	UGC(C)	3	0.19
UUA(L)	478	5.32	UCA(S)	97	2.47	UAA(*)	11	2	UGA(W)	90	1.94
UUG(L)	14	0.16	UCG(S)	0	0	UAG(*)	0	0	UGG(W)	3	0.06
CUU(L)	24	0.27	CCU(P)	60	1.98	CAU(H)	57	1.73	CGU(R)	14	1.06
CUC(L)	3	0.03	CCC(P)	10	0.33	CAC(H)	9	0.27	CGC(R)	0	0
CUA(L)	19	0.21	CCA(P)	48	1.59	CAA(Q)	60	2	CGA(R)	38	2.87
CUG(L)	1	0.01	CCG(P)	3	0.1	CAG(Q)	0	0	CGG(R)	1	0.08
AUU(I)	452	1.9	ACU(T)	67	1.91	AAU(N)	246	1.82	AGU(S)	30	0.76
AUC(I)	25	0.1	ACC(T)	6	0.17	AAC(N)	24	0.18	AGC(S)	1	0.03
AUA(M)	285	1.89	ACA(T)	67	1.91	AAA(K)	107	1.88	AGA(S)	82	2.09
AUG(M)	17	0.11	ACG(T)	0	0	AAG(K)	7	0.12	AGG(S)	0	0
GUU(V)	66	2.08	GCU(A)	65	2.39	GAU(D)	62	1.88	GGU(G)	52	1.09
GUC(V)	1	0.03	GCC(A)	3	0.11	GAC(D)	4	0.12	GGC(G)	1	0.02
GUA(V)	56	1.76	GCA(A)	39	1.43	GAA(E)	66	1.83	GGA(G)	126	2.65
GUG(V)	4	0.13	GCG(A)	2	0.07	GAG(E)	6	0.17	GGG(G)	11	0.23

### Transfer RNA and ribosomal RNA genes

*A*. *rubiginosa* and *R*. *menciana* both contain 22 tRNAs. Eight of these tRNAs (*trnQ*, *trnC*, *trnY*, *trnF*, *trnH*, *trnP*, *trnL*(CUN), and *trnV*) are coded with the minority-strand, while the remaining 14 tRNA genes are encoded by the majority-strand in *A*. *rubiginosa* and *R*. *menciana* (Table 3). The total length of the 22 tRNAs in the mt genome of *A*. *rubiginosa* is 1461 bp, and their A + T content is 81.5%. Similarly, the total length of the 22 tRNAs in the mt genome of *R*. *menciana* is 1460 bp and their A + T content is 81.8%. The AT skew is slightly positive and the GC skew is negative in the 22 tRNAs of *A*. *rubiginosa* and *R*. *menciana* (Table 4). The *rrnL* and *rrnS* genes of *A*. *rubiginosa* and *R*. *menciana* are located between *trnL1*(CUN) and *trnV* and between *trnV* and the A + T-rich region, respectively. The A + T content of the two rRNA genes is 84.7% in *A*. *rubiginosa*, which is lower than that of *R*. *menciana* (85.9%) (Table 4).

### A + T-rich region

The A + T-rich regions of *A*. *rubiginosa* and *R*. *menciana* are located between *rrnS* and *trnM* and were 399 bp and 604 bp long, respectively. The A + T-rich regions contain 92.2% and 94.0% A + T contents in *A*. *rubiginosa* and *R*. *menciana*, respectively, which were the highest across the studied mt genomes (Table 4). The AT skew and GC skew of *A*. *rubiginosa* are −0.054 and −0.097, indicating an obvious bias towards the use of T and C. However, in the *R*. *menciana* A + T-rich region, AT skew is −0.011 and the number of G and C is the same, meaning that T is more abundant than A and that the usage of G and C is equal. Several conserved structures found in other bombicoid species mt genomes are also observed in the A + T-rich regions of *A*. *rubiginosa* and *R*. *menciana*. The conserved “ATAGA + poly T” motif is located downstream of the *rrnS* gene in the A + T-rich region of *A*. *rubiginosa* and *R*. *menciana*, which may represent the origin of minority or light strand replication^31^, and is conserved in lepidopteran mt genomes. Multiple tandem repeat elements are typically present in the A + T-rich region of most insects. Only one tandem repeat was found in the *A*. *rubiginosa* mt genome (Fig. S1). We identified two tandem repeats elements in the A + T-rich region of *R*. *menciana* (Fig. S2).

The mt genome of *R*. *menciana* has been previously sequenced, and two complete mt genomes of the species are available^35, 36^. However, in the present study, there was a difference of approximately 300 nt in the length of the mt genome of *R*. *menciana* compared to the two published sequences^35, 36^. The excess 300 nt of *R*. *menciana* in the present study mainly arose from the upper area of the A + T-rich region (Fig. S2). The A + T-rich regions of the *R*. *menciana* (Ankang Shaanxi) and *R*. *menciana* (Korea) mt genomes were identical. The length of tandem repeats of the A + T-rich region of *R*. *menciana* in this study was greater than the two published sequences.

### Phylogenetic analysis

**Figure 5:**
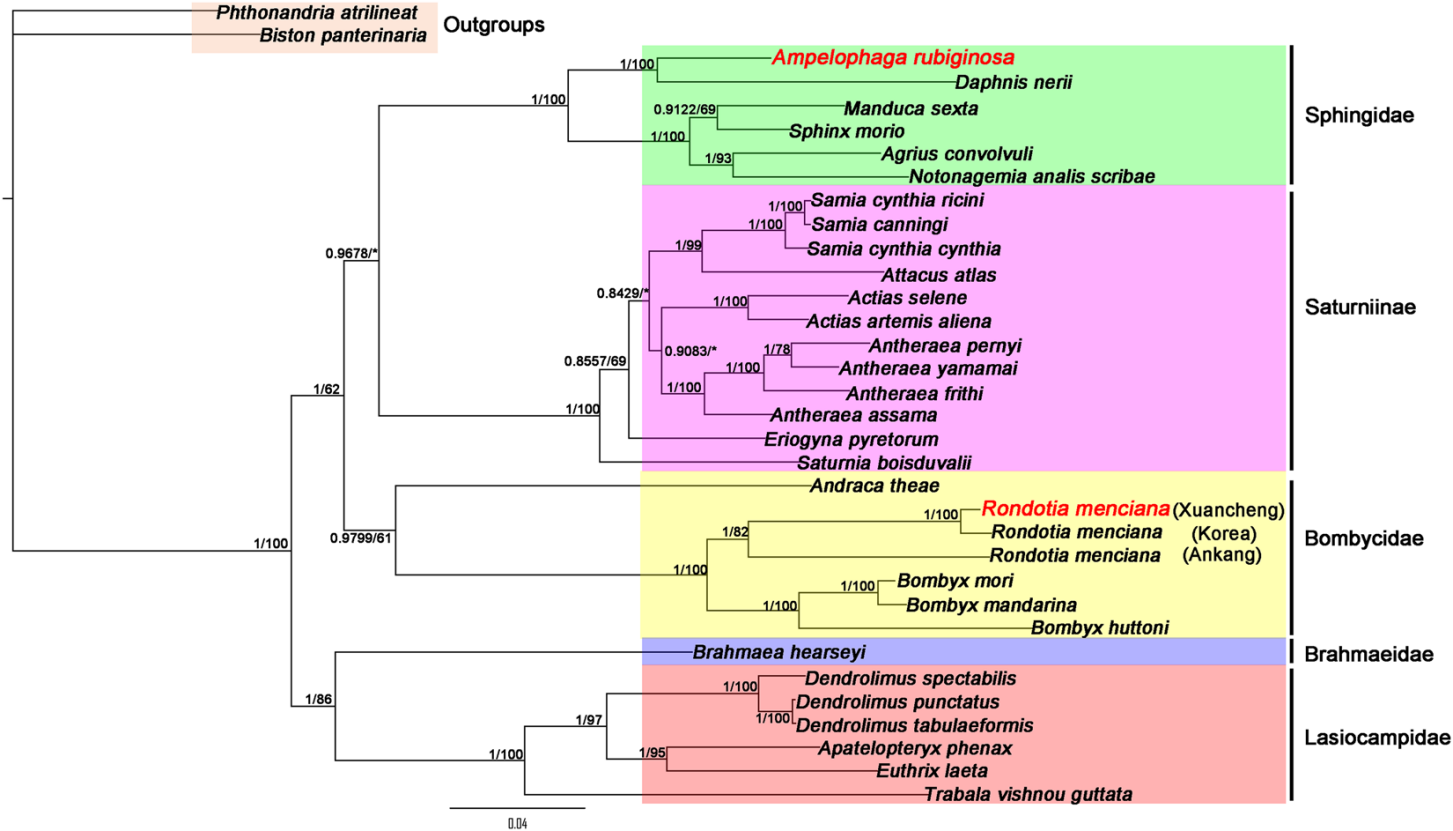
Phylogenetic tree derived for Bombycoidea using BI and ML analyses based on amino acid sequences and using MAFFT for alignment. Bayesian posterior probability (BPP) and bootstrap values (BP) of each node are shown as BPP/BP, with maxima of 1.00/100.

**Figure 6:**
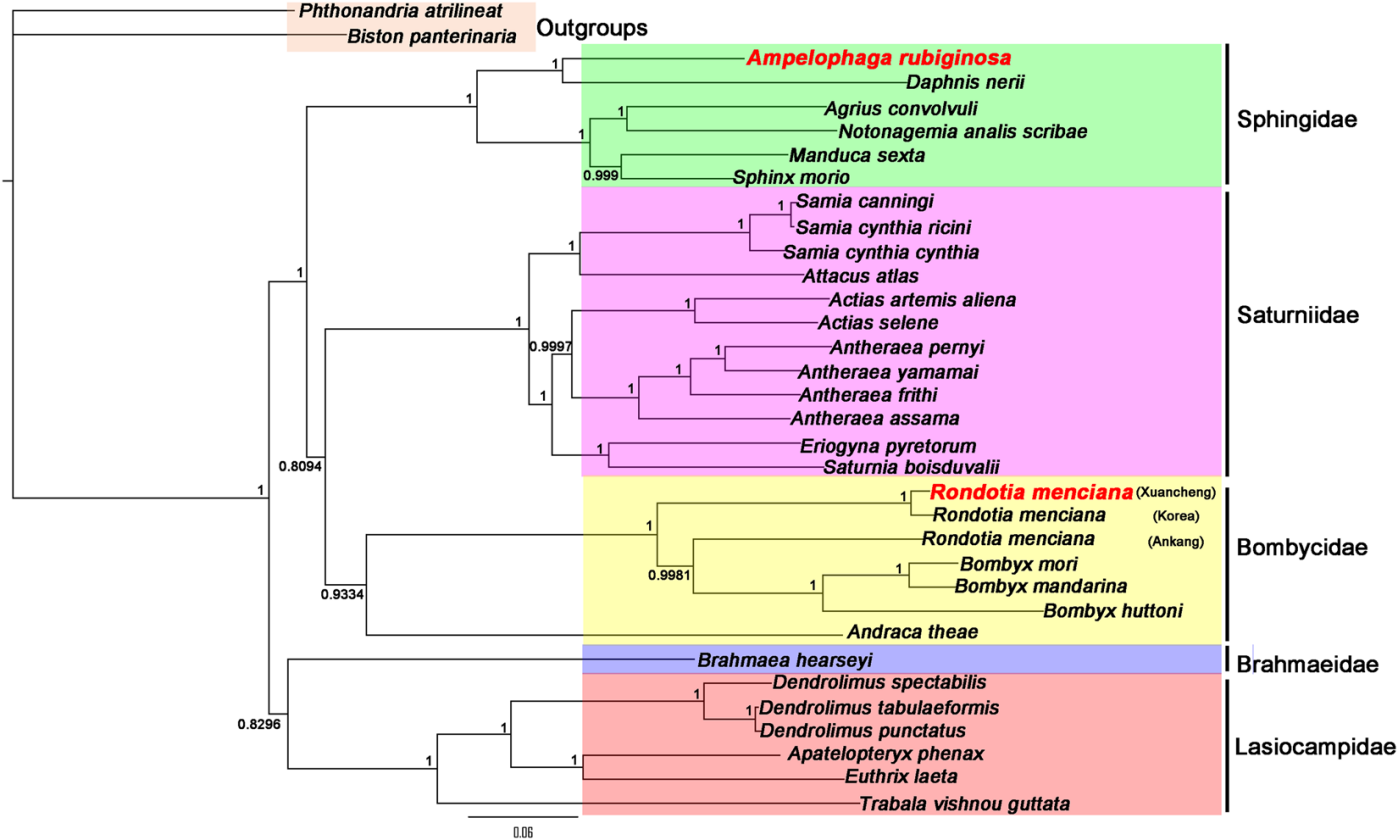
Phylogenetic tree derived for Bombycoidea using BI analysis based on nucleotide sequences using MAFFT for alignment.

**Figure 7:**
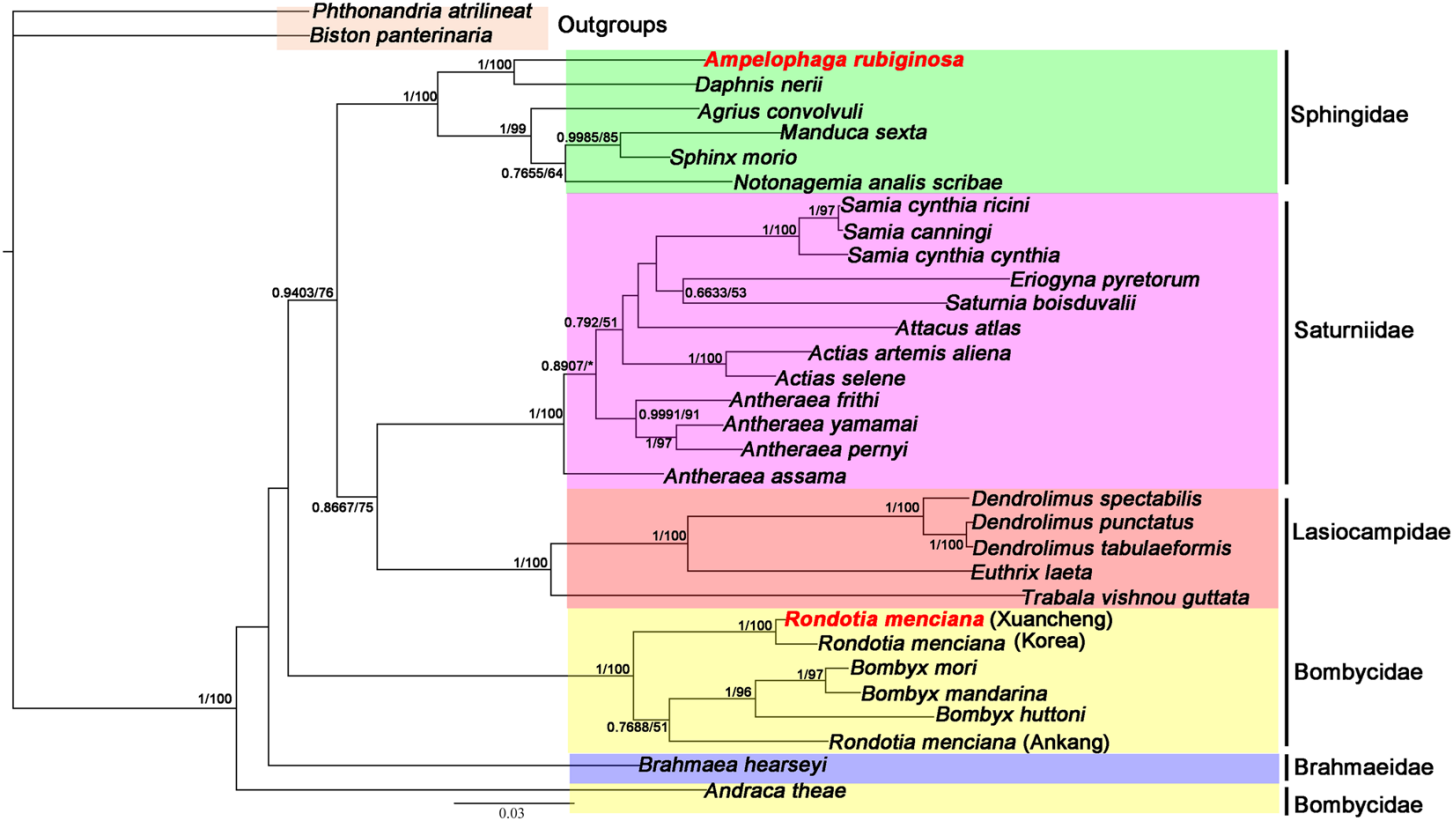
Phylogenetic tree derived for Bombycoidea using BI and ML analyses based on 16S ribosomal RNA and 12S ribosomal RNA sequences of 33 species (there are no 16S ribosomal RNA and 12S ribosomal RNA sequences in the *Apatelopteryx phenax* (KJ508055)). Bayesian posterior probability (BPP) and bootstrap value (BP) of each node are shown as BPP/BP, with maxima of 1.00/100.

Phylogenetic analyses were based on sequences of 13 PCGs of 34 mt genomes using two methods (BI and ML) and alignments performed by MAFFT. *B*. *panterinaria* and *P*. *atrilineata* were used as outgroups. Thirty bombycoid species mt genomes that were downloaded from GenBank (plus *A*. *rubiginosa* and *R*. *menciana*) represent five families belonging to the Bombycoidea: Bombycidae, Lasiocampidae, Saturniidae, Brahmaeidae and Sphingidae. It is obvious that *A*. *rubiginosa* and *Daphnis nerii* ^37^ are clustered on one branch in the phylogenetic tree with high nodal support values. The analyses show that *A*. *rubiginosa* belongs in the Sphingidae family. The three phylogenetic trees consistently showed that *R*. *menciana* from Ankang was remarkably different from those of Korea and Xuancheng. The bombycid species were *Andraca theae* + ((*R*. *menciana* (Ankang)^35^ + (*R*. *menciana* (Xuancheng) + *R*. *menciana* (Korea)^36^)) + (*B*. *huttoni* + (*B*. *mandarina* ^38^ + *B*. *mori*))), indicating that *R*. *menciana* belongs in the Bombycidae family (Figs 5, 6 and 7).

A problem remains with the phylogenetic relationships of families among the Bombycoidea in our study. The phylogenetic trees based on ML and BI analyses of amino acid sequences showed that the phylogenetic relationships were (Lasiocampidae + Brahmaeidae) + (Bombycidae + (Sphingidae + Saturniidae)) (Fig. 5), which is similar to some past studies^10, 39^. However, the phylogenetic tree based on BI analysis of nucleotide sequences showed that the phylogenetic relationships were (Lasiocampidae + Brahmaeidae) + (Sphingidae + (Bombycidae + Saturniidae)) (Fig. 6). The phylogenetic relationships of families in our study (Figs 5, 6 and 7) differ from the findings of other previous studies, where the families group as Lasiocampidae + (Saturniidae + (Bombycidae + Sphingidae))^40^. The reason for these differences may be the incorporation of complete mt genomes^39^. The relationships in the Bombycoidea remain unsettled. More mt genomes from Bombycoidea insects are required to resolve the positions of Bombycoidea in the future.

## Electronic supplementary material

SUPPLEMENTARY
