# Supplementary material for: Mitochondrial Genomes of Two Bombycoidea Insects and Implications for Their Phylogeny
Source: Sci Rep. 2017 Jul 26;7:6544. doi: 10.1038/s41598-017-06930-5 (PMC5529375; doi:10.1038/s41598-017-06930-5)

Mitochondrial Genomes of Two Bombycoidea Insects and Implications for Their Phylogeny

Zhao-Zhe Xin, Yu-Liu, Xiao-Yu Zhu, Ying Wang, Hua-Bin Zhang, Dai-Zhen Zhang, Chun-Lin Zhou, Bo-Ping Tang*, Qiu-Ning Liu*

Fig. S1 Features present in the A+T-rich region of the *A. Rubiginosa* mt genome.

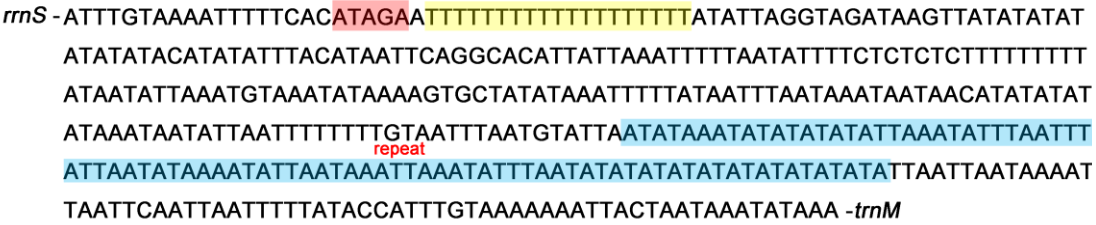

Fig. S2 Alignments of the A+T-rich region of *R. menciana* (Xuancheng Anhui, in this study), *R. menciana* (Ankang Shaanxi) and *R. menciana* (Korea).

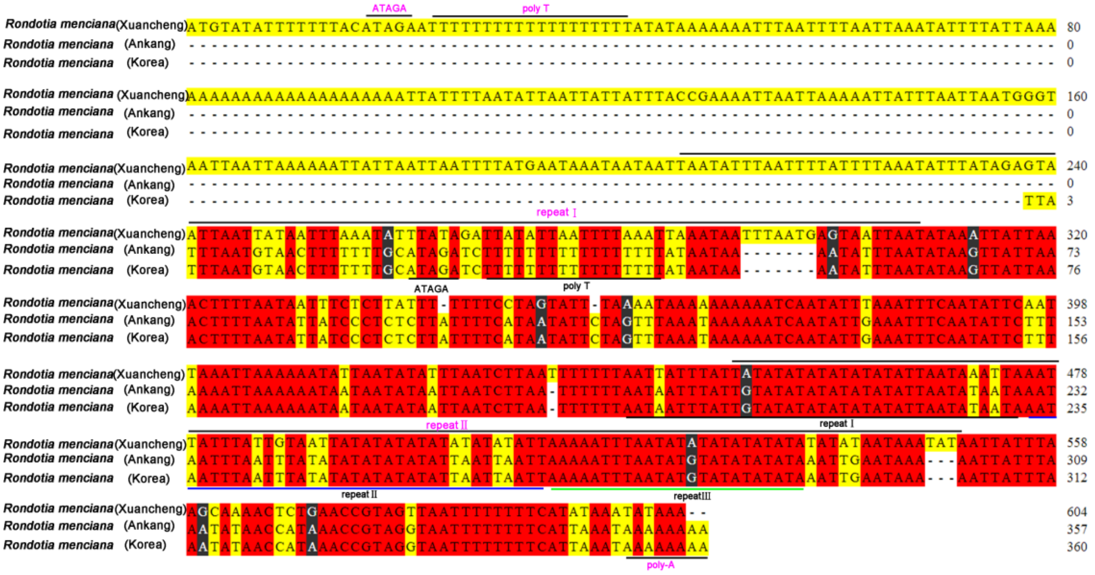

Supplement: Supplementary file 1 — SUPPLEMENTARY [file 41598_2017_6930_MOESM1_ESM.pdf]
